# Readiness for inter-professional education among healthcare professions students in Balochistan, Pakistan

**DOI:** 10.12669/pjms.41.4.10850

**Published:** 2025-04

**Authors:** Sana Shah, Ahsan Sethi, Almas Khattak, Syed Muhammad Junaid

**Affiliations:** 1 Sana Shah, BDS, MHPE. Medical Education Department, Islam Medical College, Sialkot, Pakistan; 2 Ahsan Sethi, BDS, MPH, MMEd (UK), PhD (UK) College of Health Sciences, QU Health, Qatar University, Doha, Qatar; 3 Almas Khattak, MBBS, MPH. Department of Community Medicine, Northwest School of Medicine, Peshawar, Pakistan; 4 Syed Muhammad Junaid, BDS, M.Sc., MHPE. Medical Education Department, Gandhara University, Peshawar, Pakistan

**Keywords:** Interprofessional learning, Medical, Nursing, Pharmacy, Physical therapy, Undergraduate Students

## Abstract

**Objectives:**

The study aimed to assess the readiness of undergraduate healthcare students in Balochistan, Pakistan’s regarding Interprofessional Education (IPE). It also compared readiness across disciplines, academic years, and institutional settings

**Methods:**

This analytical cross-sectional study was conducted among 374 undergraduate students from medical, nursing, physical therapy, and pharmacy programs across various institutions in Balochistan from March, 2021 to August, 2021. Data was collected using the “Readiness for Interprofessional Learning Scale” (RIPLS) to measure students’ preparedness for IPE. Statistical analyses, including t-tests and ANOVA, were performed to examine differences in readiness based on gender, institutional setting, discipline, and academic year.

**Results:**

The total mean RIPLS score was 40.07 (SD=9.323), with subscale scores for teamwork and collaboration (15.89), negative professional identity (9.59), positive professional identity (7.48), and roles and responsibilities (7.10). No gender differences were found in RIPLS subscales, but private institution students had more negative professional identity perceptions (p=0.018). Significant differences in teamwork and collaboration (p<0.001) and positive professional identity (p=0.001) were observed across disciplines, with pharmacy students showing the highest scores. Year V students showed the highest readiness for teamwork (p<0.001) and Year III students had the highest positive professional identity (p=0.034).

**Conclusion:**

The study revealed an overall low level of readiness for IPE among healthcare students in Balochistan, with notable variations based on discipline and academic year. To improve IPE readiness, there is a need for enhanced integration of IPE principles into the curriculum, increased interprofessional interactions, and a focus on addressing the challenges specific to the region. Implementing these changes could better prepare students for collaborative practice and improve patient care outcomes.

## INTRODUCTION

Interprofessional Education (IPE) involves multiple professionals learning from and with each other to enhance their ability to collaborate effectively and improve health outcomes.^1^ This educational approach is crucial for preparing future healthcare professionals to work effectively within interdisciplinary teams, which is essential for delivering patient-centered care in today’s complex healthcare environment.^2^ IPE fosters essential collaborative skills, enhances communication, and improves patient safety and overall health outcomes.^3^ It also supports a holistic approach to patient care, helping students develop a strong professional identity while understanding the roles and responsibilities of other healthcare professionals.^4^ The benefits of IPE extend beyond improved teamwork and communication. It contributes to a supportive work environment, reducing stress and burnout among healthcare professionals, and enhancing job satisfaction and retention.^5^

Given the evolving complexities of healthcare, it is increasingly recognized that no single profession can meet all the needs of a patient.^6^ Therefore, the ability to work effectively as part of a healthcare team is paramount. IPE provides the foundational skills necessary for this collaborative practice, enabling students to engage in shared decision-making and problem-solving.^7^ By learning alongside peers from different disciplines, students gain insights into the diverse perspectives and expertise each profession brings, fostering mutual respect and a shared commitment to patient care.^8^ These interactions are critical in building the competencies required for integrated, efficient, and high-quality healthcare delivery, ultimately leading to better patient outcomes and a more cohesive healthcare system.

Despite its numerous advantages, the implementation of Interprofessional Education (IPE) poses significant challenges^9^ especially in resource-constrained regions. Introducing IPE into professional curricula is a complex process that requires thorough evaluation to guide educators in assessing the quality of learning and ensuring that students are prepared for collaborative practice.^9^,^10^ While IPE has gained traction and integration globally, its implementation in Pakistan remains in its infancy and is progressing slowly.^11^-^13^ This is particularly true in Balochistan, which is one of Pakistan’s largest but most resource-constrained regions. In Balochistan, the readiness of healthcare students for IPE has not been studied, leaving a critical gap in understanding how prepared these students are to engage in interprofessional collaboration.

The need to assess IPE readiness is especially relevant in Balochistan, which hosts various healthcare training programs, including those for medicine, nursing, and applied medical sciences. Graduates from these programs are expected to collaborate in healthcare teams to deliver comprehensive, patient-centered care in hospital settings. However, initiatives to prepare these students for such roles are still in their early stages. Understanding the specific needs of these learners is crucial to developing IPE educational activities that are systematic, evidence-based, and tailored to both the students and the distinct challenges of their learning environments.

Moreover, differences in readiness for IPE may exist based on gender, profession, and other demographic factors, which could influence the effectiveness of IPE implementation. Identifying these differences is essential to address the diverse needs of students and create more inclusive and effective interprofessional learning environments.

Therefore, this study objective was address the following research questions: What are the readiness levels for Interprofessional Education among undergraduate healthcare students in Balochistan, Pakistan? What are the differences in readiness across gender, institutional setting, and disciplines? By examining these differences, the study seeks to provide valuable insights to inform the development and implementation of effective IPE programs. This will help ensure that healthcare students in Balochistan are adequately prepared to meet the demands of collaborative practice in their future careers.

## METHODS

A multi-institutional descriptive cross-sectional study was conducted from March to August 2021 in various public and private health professions education institutions in Balochistan, Pakistan, including medical, nursing, pharmacy, and physiotherapy schools. The institutions were selected to represent the diversity of health professions education within the province, capturing a wide range of educational settings and practices.

### Ethical considerations:

Ethical approval for the study was obtained from the Institutional Review Board (IRB) of QIMS (Reference Number: 900/QIMS/ERC/000102, dated March 1, 2021) and was subsequently shared with the participating colleges to ensure adherence to ethical guidelines. Informed consent was obtained from all participants prior to their inclusion in the study. This process ensured their voluntary participation and guaranteed the confidentiality of their responses, as explicitly stated in the questionnaire.

### Sample:

The study targeted undergraduate students enrolled in the medical, nursing, physical therapy, and Doctor of Pharmacy programs. A non-probability convenience sampling technique was employed to select participants from various study years (first to final year) across both public and private institutions, ensuring a representative sample within each department. The required sample size was calculated as 385 participants using the WHO sample size calculator, accounting for a 5% margin of error, a 95% confidence interval, and an estimated 50% unknown prevalence. Ultimately, 391 students participated in the study.

### Sampling Procedure:

Students from different academic years within each department were approached both on-campus and through WhatsApp student groups. The research team engaged students by visiting campuses and sharing Google Form links in the WhatsApp groups, which outlined the study’s purpose and invited voluntary participation.

### Data Collection Tool:

The “Readiness for Interprofessional Learning Scale” (RIPLS), developed by Parsell and Bligh^14^ was used to assess students’ preparedness for interprofessional education. RIPLS is a widely recognized tool designed to evaluate attitudes towards teamwork and collaboration, professional identity (both positive and negative), and understanding of roles and responsibilities within interprofessional settings.^15^ Despite some ongoing debates about its validity RIPLS was chosen due to its established use in similar studies, which facilitates comparability.^16^ The scale consists of 19 items rated on a five-point Likert scale, ranging from one (strongly disagree) to five (strongly agree), with a maximum possible score of 95.

In our study, we established cutoff scores to classify students’ readiness for interprofessional learning into three categories: scores above 72 indicated good readiness, scores between 42 and 72 indicated fair readiness, and scores below 42 indicated poor readiness. This approach was informed by reviewing the literature^17^ and expert consensus (n=3), providing a better understanding of students’ readiness for IPE.

### Data analysis:

RIPLS scores were analyzed to determine differences in interprofessional learning readiness among students based on gender, institutional setting (public vs. private), discipline (medicine, nursing, pharmacy, physical therapy), and academic year. Independent sample t-tests were used to compare scores between two groups (e.g., gender differences), while one-way ANOVA was applied to compare scores across more than two groups (e.g., different disciplines and academic years). Post hoc tests were conducted following ANOVA to identify specific group differences. Statistical significance was set at p < 0.05.

## RESULTS

The total mean score of participants on the RIPLS scale was 40.07 (SD=9.323) with a minimum score of 19 and a maximum of 78. Table-I shows the relevant results. The demographic data shows that the average age of the subjects was 21.92 (SD=2.032) with a minimum age of 18 and a maximum of 29 years. The gender distribution showed females to be predominant in number i.e., 53.5% compared to male students i.e., 46.5%. The majority of the students hailed from private settings (private= 56% vs public= 43.9%). The majority of the students were from medical disciplines (40.4%) followed by nursing (24.9%), physiotherapy (20.1%) and pharmacy (14.7%). Regarding the professional year of study, the highest number of students participated in the 4^th^ year of study (55.6%) Table-II. When asked if the students had previously participated in the Readiness for Inter-Professional Learning Scale (RIPLS) study, 57.5% said they had not while 42.5% said they had participated in the RIPLS before, with the majority of them having participated in Study 1-3 months before. The majority of the students reported having no previous exposure to interprofessional teaching and learning.

**Table-I:** Comparison of Mean RIPLS scale scores of different demographic groups of healthcare students

Group	N	Mean (± SD)	Mean Difference	P-value
All Participants	374	40.07 (9.323)		
Gender				
Male	174	40.1 (9.783)	0.08	0.929
Female	200	40.0 (8.928)		
Institutional Setting				
Private	210	40.32 (9.769)	0.56	0.560
Public	164	39.75 (8.737)		
Discipline				
Medical Students	151	41.64 (10.228)		
Nursing Students	93	37.16 (8.094)		0.044
Pharmacy Students	55	42.29 (9.121)		
Physiotherapy	75	38.90 (7.946)		
Professional Year				
I	36	39.16 (10.349)		
II	44	42.09 (10.711)		0.009
III	25	43.24 (12.917)		
IV	208	38.77 (7.729)		
V	61	42.27 (10.216)		

**Table-II:** Demographic Details of different strata of healthcare students.

Characteristic	N = (374)	
	Frequency (N)	Percent (%)
*Gender of participants*		
Male	174	46.5
Female	200	53.5
*Institutional Setting*		
Public	164	43.9
Private	210	56.1
*Discipline*		
Medical Students	151	40.4
Nursing Students	93	24.9
Pharmacy Students	55	14.7
Physiotherapy Students	75	20.1
*Professional Year*		
I	36	9.6
II	44	11.8
III	25	6.7
IV	208	55.6
V	61	16.3

The different subscale scores of Readiness for Inter-Professional Learning areas were reported showing mean scores of 15.89, 9.59, 7.48 and 7.10 for teamwork and collaboration, negative professional identity, positive professional identity and roles and responsibilities respectively Table-III. The subscale scores of different areas of RIPLS were compared by gender, institutional setting, discipline and professional year. Gender wises there were no differences among the participants towards teamwork and collaboration (P value 0.378), negative professional ID (p-value 0.995), positive professional ID (p-value 0.213) and roles and responsibilities (p-value 0.248). Regarding the institutional setting, healthcare students from private settings were more likely to have negative perceptions about their professional identity compared to public setting students (P value 0.018).

**Table-III:** Mean RIPLS Subscale Scores of Healthcare Students.

Subscale	N	Mean	SD
Teamwork and Collaboration	374	15.89	5.865
Negative Professional Identity	374	9.59	3.224
Positive Professional Identity	374	7.48	2.890
Roles and Responsibilities	374	7.10	2.090

There were no differences in their perception towards teamwork and collaboration (p-value 0.658), positive professional ID (0.925) and roles and responsibilities (p-value 0.740). When the scores of different healthcare disciplines were compared, the students showed significant differences in their readiness towards teamwork and collaboration (p-value <0.001, Pharmacy > Medical > Physiotherapy > Nursing) and positive professional identity (p-value 0.001, Pharmacy > Physiotherapy > Medical > Nursing). Significant findings were observed among students across different professional years in terms of their readiness for teamwork and collaboration (p-value < 0.001, with Year V demonstrating the highest readiness followed by Year-III, Year-II, Year-IV, and Year-I) as well as positive professional identity (p-value 0.034, with Year III exhibiting the highest positive identity followed by Year IV, Year V, Year II, and Year I) Table-IV. The individual items of the Readiness for Inter-Professional Learning scale were also analyzed reporting their mean scores.

**Table-IV:** Comparison of Mean RIPLS Subscale Scores of different demographic groups of Healthcare Students.

Group	N	MEAN (± SD)	Mean Difference	P-value
*Teamwork and Collaboration*				
All Participants	374	40.07 (9.323)		
*Gender*				
Male	174	15.60 (6.070)	-0.536	0.378
Female	200	16.14 (5.684)		
*Institutional Setting*				
Private	210	15.77 (6.151)	-0.271	0.658
Public	164	16.04 (5.492)		
Discipline				
Medical Students	151	16.16(6.700)		
Nursing Students	93	14.65 (4.778)		< 0.001
Pharmacy Students	55	17.63 (6.207)		
Physiotherapy	75	15.58 (4.632)		
Professional Year				
I	36	14.86 (5.870)		
II	44	15.95 (6.840)		<0.001
III	25	17.04 (8.696)		
IV	208	15.39 (4.643)		
V	61	17.67 (7.093)		
*Negative Professional Identity*				
All Participants	374	40.07 (9.323)		
*Gender*				
Male	174	9.59 (3.154)	-0.002	0.995
Female	200	9.60 (3.291)		
*Institutional Setting*				
Private	210	9.94 (3.101)	0.795	0.018
Public	164	9.15 (3.331)		
*Discipline*				
Medical Students	151	10.60(3.028)		
Nursing Students	93	8.90 (3.165)		0.197
Pharmacy Students	55	9.38 (3.429)		
Physiotherapy	75	8.58 (3.000)		
*Professional Year*				
I	36	10.11 (3.631)		
II	44	11.25 (2.894)		0.413
III	25	10.88 (3.192)		
IV	208	9.00 (3.045)		
V	61	9.62 (3.282)		
*Positive Professional Identity*				
All Participants	374	40.07 (9.323)		
Gender				
Male	174	7.68 (3.194)	0.373	0.213
Female	200	7.31 (2.593)		
*Institutional Setting*				
Private	210	7.47 (3.102)	-0.028	0.925
Public	164	7.50 (2.603)		
*Discipline*				
Medical Students	151	7.47(3.403)		
Nursing Students	93	7.08 (2.496)		0.001
Pharmacy Students	55	7.80 (2.422)		
Physiotherapy	75	7.76 (2.503)		
*Professional Year*				
I	36	7.08 (3.500)		
II	44	7.20(3.427)		0.034
III	25	7.64 (3.828)		
IV	208	7.57 (2.510)		
V	61	7.55 (2.929)		
*Roles and Responsibilities*				
All Participants	374	40.07 (9.323)		
Gender				
Male	174	7.23 (2.100)	0.250	0.248
Female	200	6.98 (2.080)		
*Institutional Setting*				
Private	210	7.13 (2.116)	0.072	0.740
Public	164	7.06 (2.062)		
*Discipline*				
Medical Students	151	7.39(2.103)		
Nursing Students	93	6.51 (1.937)		0.881
Pharmacy Students	55	7.47 (2.209)		
Physiotherapy	75	6.97 (2.026)		
*Professional Year*				
I	36	7.11 (2.148)		
II	44	7.68(1.997)		0.282
III	25	7.68 (2.65)		
IV	208	6.81 (1.985)		
V	61	7.42 (2.101)		

## DISCUSSION

Our study aimed to explore the readiness for interprofessional education (IPE) among healthcare students in Baluchistan, a region where such research has been notably lacking. The findings of our study indicate a mean Readiness for Interprofessional Learning Scale (RIPLS) low score of 40.7**,** reflecting poor readiness towards IPE among undergraduate students. This contrasts with earlier studies in Pakistan^11^ and that of the developed countries^18^-^21^ that showed higher scores and increase readiness for interprofessional learning. The lower mean score in Baluchistan may be attributed to the conventional, segregated instructional models prevalent in healthcare education, which limit interaction between students from different disciplines. Consequently, students may undervalue the importance of collaboration, impacting their readiness for IPE.

Our analysis highlights significant differences in RIPLS subscale scores across demographic groups. Notably, pharmacy students demonstrated higher levels of readiness for teamwork and collaboration compared to their counterparts in medicine, nursing, and physiotherapy, with nursing students showing the lower scores and decreased readiness This discrepancy suggests a potential gap in how different disciplines prepare their students for collaborative practice. Furthermore, readiness for IPE improves as students advance through their academic years, indicating that more exposure and integration of IPE concepts throughout the curriculum could be beneficial. In contrast, negative and positive professional identity scores show minimal variation across gender and institutional settings, though there is a significant difference in negative professional identity between private and public institutions. Notably, professional identity scores are higher in earlier years for positive professional identity, suggesting that early professional socialization might impact future readiness.

Another contributing factor to the low readiness could be the lack of awareness regarding the roles and responsibilities of various healthcare professionals. Students with minimal exposure to other disciplines may not fully appreciate each profession’s value to the healthcare team, leading to reduced engagement in interprofessional learning.^22^ To address this, integrating IPE into the undergraduate curriculum is crucial. Early orientation and structured IPE activities can enhance students’ understanding of interprofessional collaboration, improving leadership skills and communication, ultimately resulting in better patient safety outcomes.

Participants’ perceived lack of skills for effective interprofessional collaboration, such as communication, teamwork, and leadership, further highlights the need for formal training in these areas within healthcare curricula. The absence of institutional support and resources to implement IPE programs also contributes to students’ hesitation to participate^22^ This underscores the need for healthcare educators and institutions to prioritize IPE in their strategies, providing necessary resources and creating an environment that encourages interprofessional learning.

A comparative analysis revealed significant differences in attitudes across disciplines, with nursing students showing the lowest readiness, despite their critical role in healthcare teams. This is contradictory to previous literature where nursing students often demonstrate a higher level of readiness for IPE compared to students from other healthcare profession.^23^ In contrast, pharmacy students exhibited a relatively positive attitude towards IPE, suggesting better awareness of their collaborative role. Furthermore, readiness varied significantly across academic years, with higher scores among senior students, emphasizing the importance of early engagement in IPE to prepare students for interprofessional practice.^23^-^24^

### Limitations:

This study excluded dental and allied health schools due to limited representation, with no concrete evidence or census data to support this exclusion. The cross-sectional design only captures readiness at one point in time, missing potential changes or intervention effects. The sample is region-specific, limiting broader applicability, and self-reported data may introduce biases. Additionally, the study didn’t explore the factors influencing IPE readiness across disciplines or academic years. These limitations highlight areas for future research.

## CONCLUSION

This study examined the readiness for interprofessional education (IPE) among healthcare students. The findings reveal an overall poor readiness for IPE among students, with significant differences based on discipline and academic year. Nursing students exhibited lower readiness compared to pharmacy students, and senior students generally demonstrated higher preparedness than their junior counterparts. The low readiness is likely influenced by limited awareness of other healthcare professionals’ roles and the absence of structured IPE activities in the curriculum. These factors underscore the need for early integration of IPE into healthcare education to enhance students’ collaborative skills and improve patient care outcomes. Educators should focus on increasing interprofessional interactions and providing comprehensive training to better prepare students for collaborative practice.

### Recommendations:

Future research could explore longitudinal studies to track students’ evolving readiness for IPE and the long-term impact of IPE integration on teamwork, communication, and patient care. Comparing regional and cultural influences on IPE perceptions, especially in Balochistan, could offer valuable insights. Additionally, investigating faculty training in IPE and its effect on students’ readiness would help improve IPE practices and enhance collaborative healthcare in resource-constrained areas.

### Authors’ Contribution:

**SS and AS:** Conceived and designed the study, and wrote the first draft.

**SS and AK:** Literature search**,** Performed statistical analysis

**SS, SMJ and AS:** Reviewed and approved the final manuscript.

**SMJ:** Critical Review, Responsible for the accuracy of the work.

All authors have approved the final version to be published.
